# Low-cost automated cell counting module fabricated using CNC milling and soft lithography

**DOI:** 10.1016/j.ohx.2024.e00605

**Published:** 2024-11-17

**Authors:** Takanobu Takenouchi, Yuta Iijima, Kazuyo Ito, Daisuke Yoshino

**Affiliations:** a Department of Applied Physics, Graduate School of Engineering, Tokyo University of Agriculture and Technology, 2-24-16 Naka-cho, Koganei, Tokyo 184-8588, Japan; b Department of Biomedical Engineering, Graduate School of Engineering, Tokyo University of Agriculture and Technology, 2-24-16 Naka-cho, Koganei, Tokyo 184-8588, Japan; c Department of Food and Energy Systems Science, Graduate School of Bio-Applications and Systems Engineering, Tokyo University of Agriculture and Technology, 2-24-16 Naka-cho, Koganei, Tokyo 184-8588, Japan; d Division of Advanced Applied Physics, Institute of Engineering, Tokyo University of Agriculture and Technology, 2-24-16 Naka-cho, Koganei, Tokyo 184-8588, Japan

**Keywords:** Cell counting module, CNC milling, Soft lithography, Cell culture, Microfluidics, Cytometry

## Abstract

Cell counting is one of the basic and essential procedures that researchers in cell biology, bioengineering, and other related fields learn at the outset. Systems based on various measurement principles are commercially available, and each has its own advantages and disadvantages in terms of performance, cost, and footprint. Herein, we developed a cost-effective, scalable, and compact module that enables cell counting with reasonable accuracy, throughput, and sensitivity. This cell counting module had a size of 29 × 48 × 16 mm and a cost of $165 USD. The module can be assembled by simply inserting commercially available optical and electronic components into a housing printed by CNC milling and soft lithography. To take full advantage of this module, we built an automated cell counting system using open-source and commercially available development platforms. The module exhibited a measurement accuracy (*i.e.*, guaranteed accuracy in the concentration range of 0–500 cells/µL) and sorting resolution (*i.e.*, selection of particles with diameters of 5 µm and 15 µm) tolerable for cellular experiments. This low-cost and small-size module can be a sufficient replacement for a routine system in cell experiments. We anticipate our work will benefit research fields such as cell biology and bioengineering.

## Graphical abstract

**Figure:**
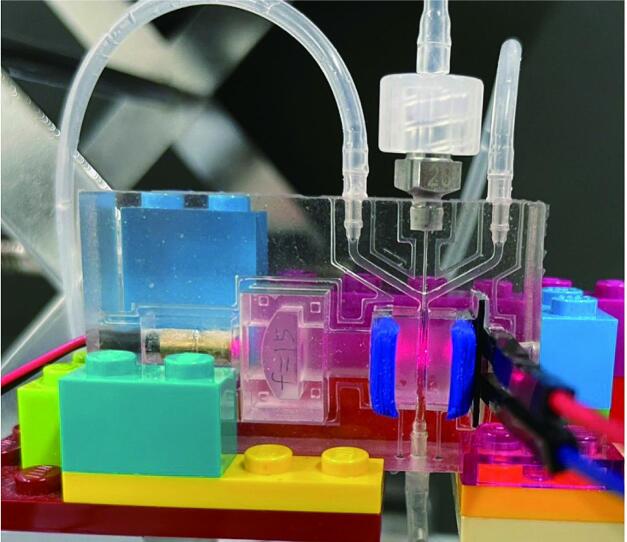

Specifications table

**Table:** 

Hardware name	Low-cost cell counting module fabricated using CNC milling and soft lithography
Subject area	• Cell biology • Biological sciences • Lab-on-a-chip • Open-source alternatives to existing infrastructure
Hardware type	• Biological sample handling and preparation
Closest commercial analog	Hemocytometer, Image cytometer (Auto cell counter)
Open source license	CC-BY-SA 4.0
Cost of hardware	26,692 JPY (∼ 165.49 USD)
Source file repository	https://data.mendeley.com/datasets/syjv86dkkk/2

## 1 Hardware in context

This paper describes the fabrication and operation of a do-it-yourself (DIY) cell counting module. Cell counting is the most basic and essential process in experiments in cell biology, bioengineering, and other related research fields. When seeding cultured cells, the number of cells (the concentration of the cell suspension) is usually adjusted using a hemocytometer, which can be calculated from the number of cells existing in a defined area. Several commercially available types have been used for different cell sizes and numbers, ranging from manual to automatic. Conventional manual hemocytometers have been the cheapest (∼ $180 USD (¥30,000 JPY)) and smallest cell counting instruments on the market [1]. However, they have significant variance in measurement accuracy, resulting from air and debris contamination, excessive or insufficient cell concentration, non-uniformity of cells located within the measurement area, and operator technique error [2], [3]. Automated cell counting systems have been introduced to solve this measurement variance. Although some improvements have been made in parts related to human error, they still need to provide a fundamental solution, and most importantly, the systems are expensive. The Coulter counter, which can measure the number and size of cells based on changes in electrical resistance, is fast, accurate, and relatively small [4]. However, it costs as much as $4,500 USD (¥400,000 ∼ ¥750,000 JPY) and is difficult to separate from other particles in the cell suspension [5]. Flow cytometers, which incorporate optical technology to provide highly sensitive and accurate measurements [6], are expensive ($50,000 USD (¥8,000,000 JPY) ∼ ), relatively large in size, and have problems of damage to cells and difficulty in maintenance. Imager-type one has become available commercially to solve those problems [7], but it is not as good a solution as one might expect regarding accuracy, size, and cost ($5,000 USD (¥600,000 JPY) ∼, plus additional costs such as dedicated consumables (*e.g.*, disposable cell slides)). Importantly, these costly systems make it difficult for the same research group to have multiple units according to the experimental environment, such as biosafety level. A low-cost, highly accurate cell counting system that is not affected by the experimental environment will satisfy users' needs.

Cell counting in flow cytometers detects changes in light intensity (forward scattered light) as cells intercept the laser light incident on the capillary. To ensure the accuracy of forward scattered light detection, the cells must be aligned in a single line. This is achieved by applying the principle of hydrodynamic constriction by forming a two-layer flow of cell suspension and sheath fluid. The flow channel should be designed so that the Reynolds number is less than 1,000 for the formation and stable maintenance of a bilayer flow. Conversely, accurate cell counting can be performed regardless of costs as long as hydrodynamic focusing is achievable. Recently, price of the optical and electronic components for light emission and detection have dropped relatively low. In device control, the advent of open-source electronic prototyping platforms (*e.g.*, Arduino) and commercial software with extensive graphical user interfaces (GUIs) (*e.g.*, LabVIEW) has lowered the hurdle to build complex scientific instruments. Furthermore, advances in rapid prototyping technologies, such as 3D printers and CNC milling machines, have eliminated the need to draft drawings and order parts from manufacturers, making it easy for scientists to give shape to what they imagine. We proposed combining these scientific and technological advances to develop an automated cell counting module that is relatively easy for users to assemble and is highly accurate. This module will enable the provision of one scientific infrastructure tailored to the experimental environment, which, we believe, contributes to the relevant biological or biomedical research field.

To this end, our current work has addressed three challenges related to automated cell counting devices. (1) modularization, (2) guarantee of a certain level of measurement accuracy, and (3) low cost. To overcome these challenges, we designed and fabricated a cell counting module using CNC milling and soft lithography techniques. Optical and electronic components were made to be insertable, and the housing and holders that hold these components are designed to be printable so that anyone can easily fabricate the module. Modularization has allowed us to improve maintainability, reduce the size, and easily adapt the system to any experimental environment. Optimization of the hydrodynamic focusing achieved cell alignment even with the millimeter-scale flow channel. This enables highly accurate optical measurements under conditions with minimal damage to cells. We further developed a low-cost, automated cell-counting module using Arduino and LabVIEW platforms. Our work has improved the maintainability and cost-effectiveness of cell-counting systems, allowing highly accurate automated cell measurements that were previously only possible in a limited number of laboratories. This will contribute to the creation of diverse research results by further facilitating day-to-day research activities in fields such as biology and bioengineering. We also hope that the developed system will be useful in situations where it is difficult to install expensive equipment, such as resource-limited environments, classrooms, and early-stage research.

## 2 Hardware description

Existing cell counting instruments are expensive, large, or inaccurate depending on the measurement principle, but we have developed an easy-to-maintain automated cell counting module that combines the best features of these instruments (Table 1). Our cell counting module is based on the highly sensitive and accurate optical technology of flow cytometers, introducing standard optical and electronic components costing only a few to several tens of dollars (several hundred to several thousand yen). The module is designed to be printable, insertable, high-yield, and connectable to other analysis modules. The module also uses only readily available, inexpensive, and off-the-shelf components (cylindrical lens, diode laser, photodiode, Arduino, etc.). We achieved a small size (29 × 48 × 16 mm), reasonable throughput (16.7 cells/sec), and low cost ($165 USD (¥26,700 JPY)) by introducing only the functions related to cell counting, so that the module can be connected to modules with other functions. Even compared to other DIY devices reported in previous studies, the module we developed, including the system for its operation, is low cost.

**Table 1:** Summary of the DIY cell counting module and the commercially available system. Symbol mark notation meanings are as follows: +, poor; ++, average; +++, good; ++++, excellent; $, low; $$, average; $$$, high.

Device type	Principle	Accuracy	Throughput	Selectivity	Maintenance	Cost [USD]	Size [mm]	Reference
This study	Optical intensity	++ ∼	+++ (16.7 cells/sec; Potential for improvement)	++ (Potential for improvement)	+++	$ (165USD)	++++ (29 × 48 × 16)	
Hemocytometer	Manual	++	++	+	+++	$	++++ (30 × 70 × 4)	Neubauer chamber, Electron Microscopy Sciences
Coulter counter	Electrical resistance	+++	+++	++	+		++ (283 × 64 × 109)	Scepter 3.0 Handheld Automated Cell Counter, Millipore
Flow cytometer	Optical intensity	++++	++++	++++	+	$	+ (400 × 580 × 430)	Attune™ Flow Cytometer, Thermo Fisher Scientific
Image cytometer	Image processing	++	+++	+++	+		++ (244 × 170 × 239)	Invitrogen™ Countess 3, Thermo Fisher Scientific
3D-printed imaging platform	Image processing	++ ∼	+++	+++	N/A	$ (745USD) Note: amount excluding syringe pump	++ (50 × 75) Information on the dimensions of the flow cell only.	Awate *et al*. *Analyst* 2021 [8]
Lab on a chip flow cytometer	Optical intensity	++++	++++	++++	N/A	(5,000USD)	++ (390 × 220 × 100)	Mohan *et a*/. BIOSTEC 2017 [9]

### 2.1 Cell counting module

The developed cell counting module has a relatively large flow channel scale ranging from 500 µm to 1 mm, so it is not quite right to call it a microfluidic device. The high aspect ratio structure required to embed inexpensive, readily available optical components makes photolithographic mold fabrication difficult. In contrast, CNC milling machines can easily cut molds with these structures. The hydrodynamic optimization described below does not cause problems with cell counting, even in millimeter-scale flow channels. These channels allow seamless connection, which is often a problem when modularizing microfluidic devices, to other modules with commercially available luer fittings (VPI116, Nordson Medical). This high modularity could be used in the future to connect with multiple fluorescence detection and sorting modules to build ultra-low-cost flow cytometers and cell sorters (probably around $1000 USD). The flow channel modules, including the cell-counting one developed in this work, provide the researcher with an easy way to obtain a miniature experimental facility by assembling the modules with the desired experimental configurations based on the base plate of LEGO® or other products.

**Fig. 1:**
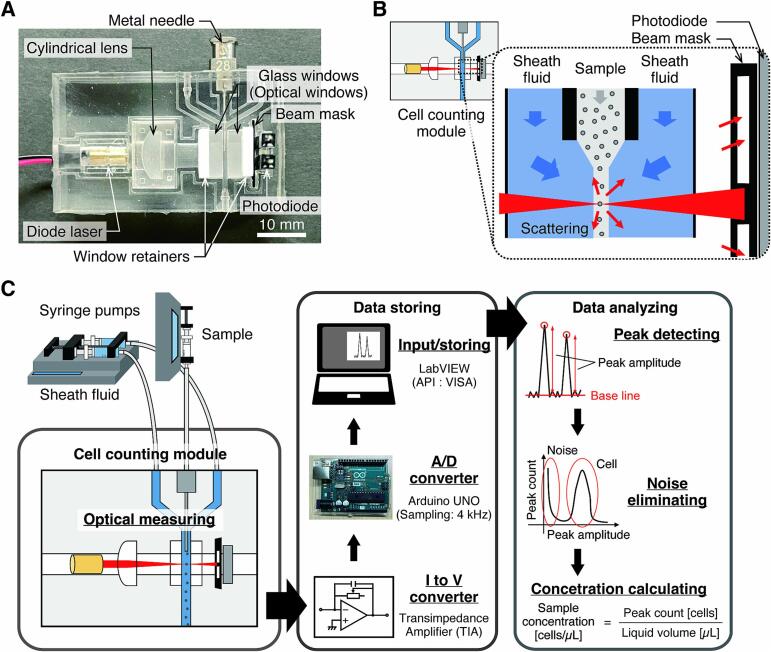
Low-cost automated cell counting system. A) Cell counting module made of PDMS housings. Optical and electronic components are insertable, and the housing and holders that hold these components are designed to be printable. B) Principle of cell counting: A forward scattered light generated when each cell flowing in a line in a channel passes through the area of laser light focusing is detected by a photodiode. C) Configuration of the cell counting system: Cell-derived signals detected in the module pass through the “data storing” and “ data analyzing” sections to calculate a sample concentration.

The current cell counting module consists of a polydimethylsiloxane (PDMS) base body with a flow channel through which the sample (cell suspension) and sheath fluid flow, and a mounting section for various optical components into which a diode laser, a photodiode, a cylindrical lens, glass windows, and a beam mask are inserted and held in place by PDMS holders or 3D-printed retainers (Fig. 1A). The flow channel has glass windows to secure the laser beam path and prevent fluid leakage. The sample is injected into the channel through a metal needle. The pumping device for the sample and sheath fluid should be pulsation free, although in most cases this is not a problem as long as a constant flow rate can be maintained. Cells flowing in a line pass through the laser focusing point one by one, scattering the light (Fig. 1B). The scattered light is detected as a current by a photodiode through a hole in the beam mask, converted to a voltage by a transimpedance amplifier (TIA), and output to a PC with an A/D converter board (Arduino in this case) (Fig. 1C). The data input to the PC is analyzed using predetermined criteria, and the number of cells is measured by calculating the scattering frequency using a program (LabVIEW in this case). The size of this module is 29 × 48 × 16 mm, which is not much larger than the commercially available hemocytometer (*e.g.*, Burker-Turk’s Counting Chamber, Erma Inc.).

### 2.2 Hydrodynamic focusing: Measurement principle

**Fig. 2:**
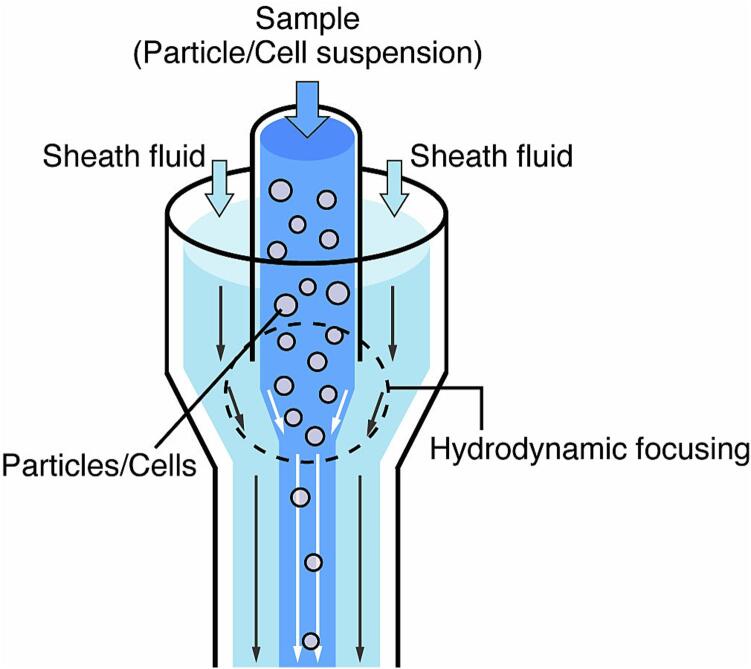
Illustration of the measurement principle of optical cell counting (*i.e.*, Hydrodynamic focusing). A two-layer flow is formed by the flow of sheath fluid in addition to the sample, and the sample flow is hydrodynamically focused.

To measure cells individually as they flow through the channel, we must align the cells in a single column and read the information from each cell in turn. Most flow cytometers utilize “hydrodynamic focusing [10],” in which the cell suspension flows at a controlled flow rate through the area confined by the cell-free sheath fluid (Fig. 2). Focusing this region to a size where only one cell can pass will reduce the variance of the forward scattering and thereby improve detection accuracy. This hydrodynamic focusing is obtained by forming a two-layer flow so that the cell suspension and the sheath fluid do not mix. Therefore, the flow channel should be designed so that the Reynolds number is less than 1000. Reynolds number *Re* (dimensionless number) is defined as follows.

Re=ρvDhμ

where ρ is the density of the fluid (kg/m^3^), µ is the viscosity (Pa·s), v is the average velocity (mm/s), and $D_{h}$ is the hydraulic diameter (mm), which is defined for a channel with a rectangular cross-section of length (a, b) (mm) by the following formula.

$$D_{h}=\frac{4ab}{2\left(a+b\right)}=\frac{2ab}{a+b}$$

The module designed and fabricated in this study is a square cross-section channel of 1 mm square. When the density of the solution is 1.0 × 10^3^ kg/m^3^, the viscosity is 1.0 × 10^-3^ Pa·s, and the average velocity is 0.5–2.0 mm/s, the Reynolds number is 0.5–2.0 indicating that the hydrodynamic focusing works.

**Fig. 3:**
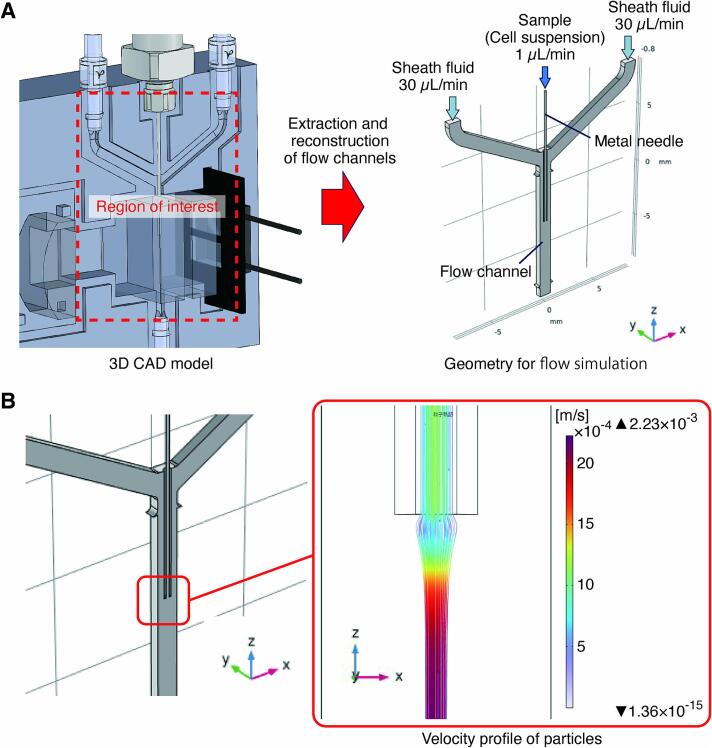
Confirmation of hydrodynamic focusing performance by numerical analysis. A) Model and boundary conditions used in the simulation. B) Estimated velocity distribution of the sample in the direction of flow.

A numerical simulation was performed to verify that the hydrodynamic focusing was effectively obtained. The laminar flow and particle tracking modules of the CAE software (COMSOL Multiphysics®, COMSOL, Inc.) were implemented into the simulation. The flow channel models were created in a 3D CAD package (SolidWorks, Dassault Systèmes SolidWorks Corp.) and imported into the CAE software (Fig. 3A). The steady-state flow field in the channel was calculated by setting the flow rate of the sample (cell suspension) to 1 µL/min and the flow rate of the sheath fluid inflowing from the left and right channels to 30 µL/min. Cells simulated as particles with a diameter of 10 µm and a density of 1050 kg/m^3^ were then injected through the sample inlet, and particle tracking was performed by time-dependent analysis. Focusing on the particle tracks (Fig. 3B), we can see that the flow is slightly widened immediately after passing through the needle tip due to pressure changes associated with the expansion of the channels but then narrows down to the center of the channel. Under this condition, the Reynolds number is Re≈1, which is below the theoretical value of the critical number for two-layer flow formation [11]. In addition, the particle velocity increases to a maximum of 2.33 mm/s after merging with the sheath fluid. This is consistent with the theoretical maximum velocity being twice the mean rate in a Poiseuille flow [12]. The particles pass within 50 µm of the channel center, confirming that the hydrodynamic focusing has been adequately implemented.

### 2.3 Scattered light detection: Measurement principle

**Fig. 4:**
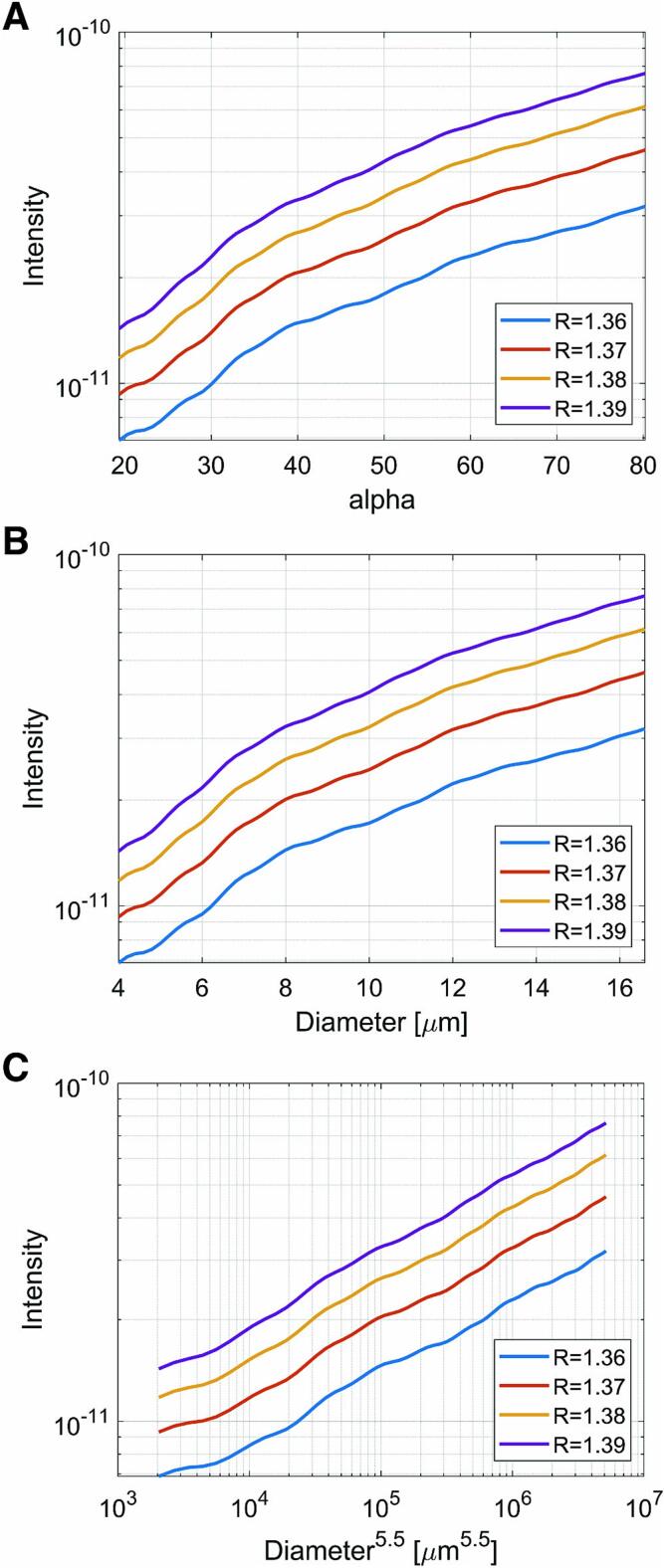
Numerically calculated forward scattered light intensity as a function of A) size parameter alpha, B) cell diameter, and C) 5.5 power of cell diameter. The coefficient R is the reflection index of the cytoplasm reported by the literature [16].

Flow cytometry detects scattered light generated when light from a laser source is focused and hits a cell. Scattered light is classified into two types: forward scattered light, which is measured coaxially with the direction of laser incidence, and side scattered light, which is measured at an angle of 90° to the direction of laser incidence [13] Forward scattered light is diffracted or refracted at the cell surface and thus refers to the size of the cell. On the other hand, side scattered light is refracted within the cell and provides information about cell morphology, nucleus, organelles, and other internal cell structures. The forward scattered light is generally used for cell counting. In this module, the laser light is focused by a cylindrical lens into a line perpendicular to the flow direction, irradiated onto cells, and its forward scattered light is detected by a photodiode after eliminating stray light with a beam mask. The size of a cell is sufficiently larger than the wavelength of the laser light. Thus, the scattering phenomenon when the light hits a cell is considered as Mie scattering [14]. Assuming that the cell is a spherical particle with a uniform internal radius a (µm), and solving Maxwell's equations with the boundary conditions on the sphere surface, the relative intensity $I(\alpha ,\theta_{1},\theta_{2})$ of the forward scattered light (relative value assuming incident light intensity of 1; dimensionless numbers) at the angle $\theta_{1}$ to $\theta_{2}$ is obtained as follows [15].

$$I\left(\alpha ,\theta_{1},\theta_{2}\right)=\frac{\lambda^{2}}{4\pi^{2}}\int_{\theta_{1}}^{\theta_{2}}\left\{i_{1}\left(\alpha ,\theta \right)+i_{2}\left(\alpha ,\theta \right)\right\}\sin\theta d\theta$$

where λ is the wavelength of the light source (nm), α(=2πa/λ) is the size parameter(dimensionless number), $i_{1}$ and $i_{2}$ are intensity functions (dimensionless numbers), $\theta_{1}$ and $\theta_{2}$ indicate the range of angles at which light is scattered in the forward direction (°), as limited by the beam mask. Here, the angles $\theta_{1}$ and $\theta_{2}$ are 7.8° and 28.6° respectively, based on the size of the prepared mask. Based on this equation and the refractive index of the cell (1.36–1.39; cytoplasm [16]), the dependence of cell size on scattering intensity $I(\alpha ,\theta_{1},\theta_{2})$ in proportion to alpha is calculated numerically for $\theta_{1}$ and $\theta_{2}$ combinations. This can be easily solved, for example, using the MATLAB scripts [17]. These results show that the scattering intensity $I(\alpha ,\theta_{1},\theta_{2})$ is proportional to the 5.5 power of the cell diameter (2a) up to about 12.4 µm (Fig. 4). Of course, actual living cells are not perfectly spherical and are affected by nuclei and intracellular organelles, but these are not considered to be major problems for the accuracy of cell counting.

**Fig. 5:**
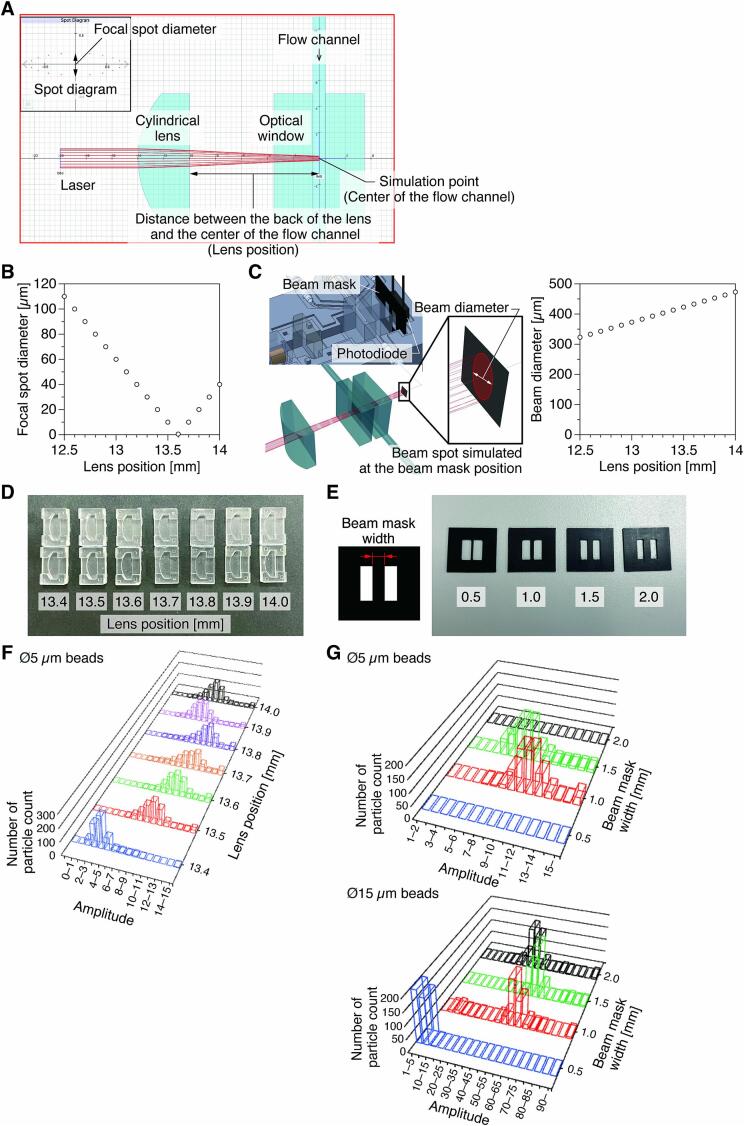
Optimization of the optical components to maximize the sensitivity of the detection of the scattered light. A) Simulation of laser beam focusing at the center of the flow channel for changing the lens position. B) Correlation between lens position and focal spot diameter of the laser beam at the center of the flow channel. C) Estimated beam diameter at the beam mask position (just in front of the photodiode) for each lens position. D) Holders that specify each lens position fabricated based on simulation results. E) Beam masks of various widths fabricated based on simulation results. Verification of the measurement sensitivity of the developed module: F) Relationship among the lens position, the signal amplitude detected by the photodiode associated with the scattered light intensity, and the number of particle counts; and G) the relationship among the beam mask width, the signal amplitude, and the number of particle counts for two different diameters of particles.

To maximize detection sensitivity, it is important to optimize the position of the cylindrical lens, which focuses the laser light, and the shape of the beam mask, which eliminates stray light. For these optimizations, we used the software RayLab [18] to determine the estimated values by ray tracing simulations and then verified their validity experimentally. The spot diameters of the collimated light (1.5 mm in diameter and 650 nm in wavelength) focused by a cylindrical lens in a channel (water, 1 mm in width) sandwiched between optical windows (N-BK7, 3 mm in thickness) were estimated at the channel center and beam mask position (Fig. 5A). The lower limit of the beam mask width was determined from the estimated spot diameter (*i.e.*, the short axis length of the focused light spot). Given that the cylindrical lens used for the module has a back focus distance of 12.5 mm and the focal length extends at the boundary between the optical window and the water, the distance between the back of the lens and the center of the flow channel (*i.e.*, the lens position) was set between 12.5–14.0 mm, and the optimum value was calculated to be 13.6 mm (Fig. 5B). The focal spot diameter under this condition was 0.50 µm. In this case, the beam diameter just in front of the photodiode was calculated to be 433 µm (Fig. 5C), and thus, a beam mask width of at least 0.5 mm is predicted to be desirable. Based on the estimation results, the lens position and beam mask width that can maximize the detection sensitivity were verified. Using the estimated values as a reference, models were fabricated for lens positions from 13.4 to 14.0 mm at 0.1 mm intervals (Fig. 5D) and for beam mask widths from 0.5 to 2.0 mm at 0.5 mm intervals (Fig. 5E).

To verify the lens position, we measured the scattered light peaks for 200 microbeads (G0500, Thermo Fisher Scientific) with a diameter of 5 µm for three times (600 beads in total). For the beam mask width verification, we used two types of microbeads, 5 µm in diameter (G0500; 600 beads in total for each mask width) and 15 µm in diameter (A4215, Thermo Fisher Scientific; 400 beads in total for each mask width). The verification results suggest that 13.6 mm and 1.5 mm are the optimal lens position and beam mask width values, respectively (Fig. 5F and G). The deviations from the simulation results are assumed to be due to input and boundary conditions that do not match the actual conditions, such as ideal parallel light.

**Features:**

• Affordability; less expensive (∼$180 USD) than commercial alternatives.
• Small footprint (29 × 48 × 16 mm).
• Modular design allows components to be reused and connected to other modules.

## 3 Design files

**Design files summary**

**Table:** 

**Design file name**	**File type**	**Open source license**	**Location of the file**
Part #3	STL file; CAD file	CC	Available with the article
Part #4	STL file; CAD file	CC	Available with the article
Part #5	STL file; CAD file	CC	Available with the article
Part #8	STL file; CAD file	CC	Available with the article
Part #9	STL file; CAD file	CC	Available with the article
Part #10	STL file; CAD file	CC	Available with the article
Part #16	STL file; CAD file	CC	Available with the article
Part #17	STL file; CAD file	CC	Available with the article
Part #18	STL file; CAD file	CC	Available with the article

**Part #3** is a beam mask with holes in specific locations so that only the scattered light enters the detector.

**Part #4** is a retainer that keeps the glass window in place, and it has a hole through which the laser light can pass.

**Part #5** is the concave side of the base body of the cell counting module, which has bumps and dents for alignment because it is used in conjunction with **Part #10**.

**Part #8** is a holder for a laser module.

**Part #9** is a holder for a cylindrical lens. The position of the lens can be adjusted by simply reshaping the holder without modifying the body.

**Part #10** is the convex side of the base body of the cell counting module, which has bumps and dents for alignment because it is used in conjunction with **Part #5**.

**Part #16** is the mold to fabricate **Part #5** and **Part #10** with soft lithography.

**Part #17** is the mold to fabricate **Part #8** with soft lithography.

**Part #18** is the mold to fabricate **Part #9** with soft lithography.

## 4 Bill of materials

**Fig. 6:**
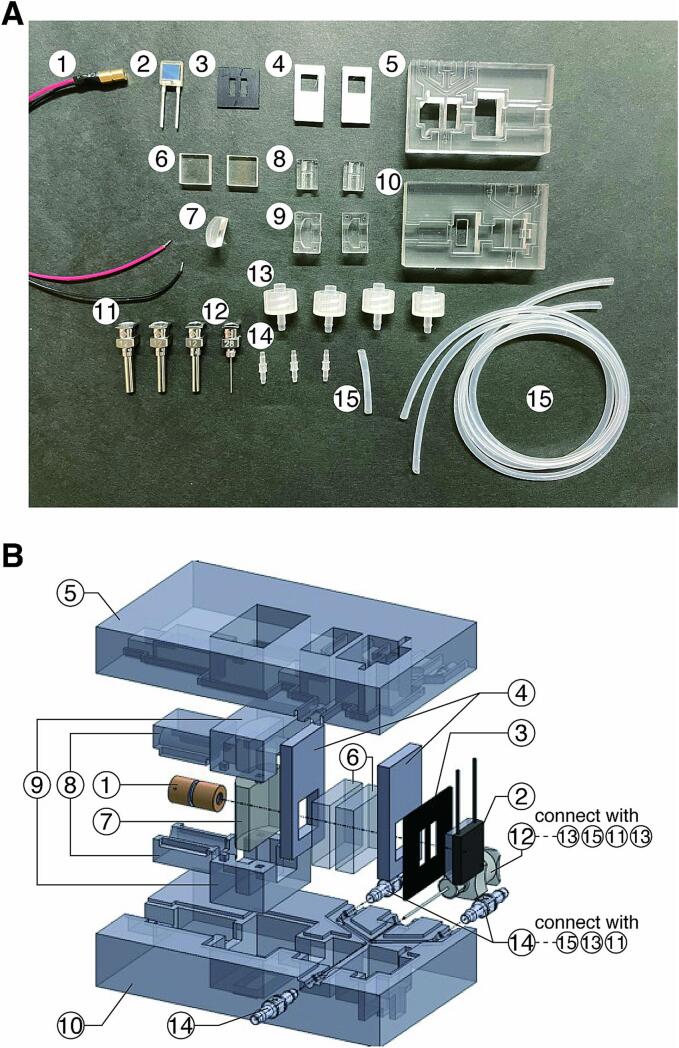
Cell counting module kit. A) Photograph of the kit. **Part #1**) Red laser module, RP650AD5-4C. **Part #2**) Si PIN photodiode, S6967. **Part #3**) CNC-milled beam mask. **Part #4**) 3D-printed window retainers. **Parts #5** and **#10**) PDMS base bodies fabricated with soft lithography. **Part #6**) Optical windows, OPB-10S03-P. **Part #7**) Cylindrical lens, CLB-1010-15PM. **Parts #8** and **#9**) Laser and lens holders fabricated with soft lithography. **Parts #11** and **#12**) Metal needles, SNA-12G-C and SNA-28G-B. **Parts #13** and **#14**) Luer fittings, VRM106 and VPI116. **Part #15**) Silicone tube, ID: 1 mm, OD: 1.5 mm. B) Assembly diagram of the module. (For interpretation of the references to colour in this figure legend, the reader is referred to the web version of this article.)

The cell counting module consists of 15 parts **(Parts #1–#15**; Fig. 6). The bill of materials also includes molds for fabricating PDMS parts (**Parts #16–18**), syringes and pumps for injecting samples and sheath fluids, and electronic components for TIA and A/D conversion.

**Bill of materials summary**

**Table:** 

**Designator**	**Component**	**Number**	**Cost per unit − JPY**	**Total cost − JPY**	**Source of materials**	**Material type**
Part #1	Red laser module, Cat No. RP650AD5-4C	1	650	650	https://akizukidenshi.com/catalog/g/g112279/	Semiconductor
Part #2	Si PIN photodiode, Cat No. S6967	1	400	400	https://akizukidenshi.com/catalog/g/g107332/	Semiconductor
Part #3	Beam mask [Polyvinyl chloride (PVC) board (220 × 300 × 1 mm)]	0.1	151/sheet	15	https://www.hazaiya.co.jp/item/14506.html	PVC
Part #4	Window retainer	2	3,600/kg	3	https://flashforge.shop-pro.jp/?mode = grp&gid = 2958637	PLA
Part #5	Concave side of the base body	1	35,530/kg	355	https://www.dow.com/en-us/pdp.sylgard-184-silicone-elastomer-kit.01064291z.html#overview	PDMS
Part #6	Optical window, Cat No. OPB-10S03-P	2	1,800	3,600	https://jp.optosigma.com/ja_jp/opb-10 s03-p.html	BK7
Part #7	Cylindrical lens, Cat No. CLB-1010-15PM	1	11,900	11,900	https://jp.optosigma.com/ja_jp/clb-1010–15 pm.html	BK7
Part #8	Laser holder	2	35,530/kg	36		PDMS
Part #9	Lens holder	2	35,530/kg	53		PDMS
Part #10	Convex side of the base body	1	35,530/kg	355		PDMS
Part #11	12G metal needle, Cat No. SNA-12G-C	3	175	525	https://www.musashi-engineering.co.jp/products/nozzle/nozzle/SNA	Metal
Part #12	28G metal needle, Cat No. SNA-28G-B	1	130	130	https://www.musashi-engineering.co.jp/products/nozzle/nozzle/SNA	Metal
Part #13	Luer fitting, Cat No. VRM106	4	110	440	https://axel.as-1.co.jp/asone/d/5–1043-01/	PP
Part #14	Luer fitting, Cat No. VPI116	3	150	450	https://axel.as-1.co.jp/asone/d/5–1047-01/	PP
Part #15	Silicone tube 1 × 1.5	1	200/m	100	https://axel.as-1.co.jp/asone/d/61–9432-28/	Silicone
Part #16	Polyacetal (POM) plate for the mold of the main body(100 × 100 × 15 mm), Cat No. PAA-100–100-15	1	990	990	https://jp.misumi-ec.com/	POM
Parts #17 and #18	POM plates for the molds of the holders for a laser module and cylindrical lens(80 × 80 × 10 mm), Cat No. PAA-80–80-10	2	860	1720	https://jp.misumi-ec.com/	POM
1 mL syringe	1 mL Terumo syringe, Cat No. SS-01 T	1	3,000/box(1 box contains 100 pcs)	30	https://www.terumo.co.jp/medical/equipment/me05.html	Other
5 mL syringe	5 mL Terumo syringe, Cat No. SS-05SZ	2	3,000/box(1 box contains 100 pcs)	60	https://www.terumo.co.jp/medical/equipment/me05.html	Other
Arduino UNO	Arduino UNO Rev3, Cat No. A000066	1	3,630	3,630	https://akizukidenshi.com/catalog/g/g107385/	Other
USB cable	USB cable A-B	1	100	100	https://akizukidenshi.com/catalog/g/g117015/	Other
Breadboard	Breadboard	1	150	150	https://akizukidenshi.com/catalog/g/g105155/	Other
Operational amplifier (Op-Amp)	Rail-to-Rail single supply operational amplifier, Cat No. NJM2737D	1	100	100	https://akizukidenshi.com/catalog/g/g108231/	Other
Potentiometer	5 MΩ potentiometer, Cat. No. 3386 K-EY5-105TR	1	50	50	https://akizukidenshi.com/catalog/g/g106116/	Other
Capacitor	0.01 µF mutilayer ceramic capacitor, Cat No. RD15W103K1HH5L	1	100	100	https://akizukidenshi.com/catalog/g/g102281/	Other
Jumper Wire	Jumper wire set, Cat No. BBJ-65	1	300	300	https://akizukidenshi.com/catalog/g/g105159/	Other
Breadboard jumper wire	Breadboard jumper wire set, Cat No. 165–011-000	1	450	450	https://akizukidenshi.com/catalog/g/g102315/	Other
Syringe pump for sample	Legato 110	1				Other
Syringe pump for sheath fluid	KDS 270	1				Other

Cost in USD were converted from JPY (exchange rate: 0.0062 USD).

Total Cost: 26,692 JPY ≈ 165.49USD.

## 5 Build instructions

The main bulk of the cell counting module (including the base body) was fabricated using a 3D printer or soft lithography using CNC-milled molds. The rest of the components were purchased online (Bill of Materials). The main bulk of PDMS is divided into two parts: the base body, which contains the flow channels and holds the components, and the component holders, which hold the lenses and lasers. Optical systems can be easily modified by changing the component holders, because the focal length of the lens changes with the wavelength of the light source (*i.e.*, chromatic aberration). The additional tools or machines required for fabrication and assembly included:

• Vacuum chamber
• Ultrasonic cleaner
• Autoclave
• Plasma cleaner

### 5.1 Hardware preparation

#### 5.1.1 CNC machining

**Fig. 7:**
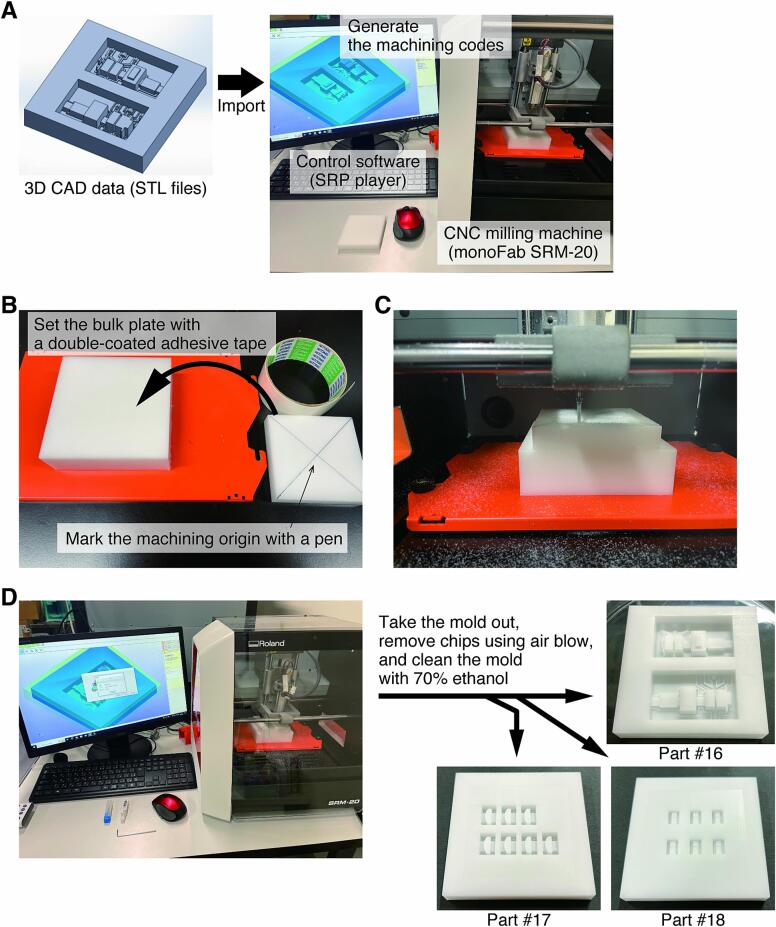
Procedures for the CNC machining of part molds for soft lithography. A) Import of 3D CAD data into the control software and generation of machining codes. B) Setting of a bulk plate on the milling machine. C) Scene of the machining process. D) Part removal and cleaning after the machining process.

Export the 3D CAD data (STL files) of the molds (**Parts #16, #17**, and **#18**) for manufacturing the base body (**Parts #5** and **#10**), laser module holder (**Part #8**), and cylindrical lens holder (**Part #9**) to the control software of the milling machine and generate the machining codes (Fig. 7A). The mold should be machined from a polyacetal bulk plate because of the mold release properties of PDMS. The CNC milling machine is not limited to a specific type. This work used a compact bench-top model (monoFab SRM-20, Roland DG). **Note:** Milling conditions are important because they affect the yield of the mold machining (see Table 2). At this point, the area to be milled should be larger than the size of the bulk plate to prevent steps from forming on the mold's top surface.

**Table 2:** Conditions for milling each part. Conditions for each CNC milling machine must be optimized because these are suitable for monofab SRM-20, Roland DG.

		Part#3	Part #16	Part #17	Part #18
Roughing process	Cutting tool (end mill)	Square	Square	Square	Square
	Blade diameter [mm]	0.5	2.0	0.8	0.8
	Spindle revolution [rpm]	7,000	7,000	7,000	7,000
	Cutting speed [mm/min]	600	900	800	800
	Depth of cut [mm]	0.2	0.25	0.2	0.2
	Path interval [mm]	0.2	0.4	0.2	0.2
	Finishing allowance [mm]	0.1	0.4	0.1	0.1
Finishing process 1	Cutting tool (end mill)	Square	Square	Square	Square
	Blade diameter [mm]	0.5	0.8	0.5	0.8
	Spindle revolution [rpm]	7,000	7,000	7,000	7,000
	Cutting speed [mm/min]	600	800	960	960
	Depth of cut [mm]	0.01	0.2	0.01	0.05
	Path interval [mm]	0.02	0.2	0.01	0.01
	Finishing allowance [mm]	0	0.2	0	0
Finishing process 2	Cutting tool (end mill)		Square		
	Blade diameter [mm]		0.8		
	Spindle revolution [rpm]		7,000		
	Cutting speed [mm/min]		800		
	Depth of cut [mm]		0.03		
	Path interval [mm]		0.03		
	Finishing allowance [mm]		0
Set the bulk plate marked with the machining origin on the milling machine (Fig. 7B).
Set the machining origin of XYZ axes of the milling machine.
Start the machining process (Fig. 7C).
Take the mold out of the machine after processing, remove chips using an air blow dust gun, and clean the mold with 70 % ethanol (Fig. 7D).
Beam mask (**Part #3**) is also manufactured by CNC milling machine. Use PVC board as material instead of polyacetal bulk plate.

#### 5.1.2 Soft lithography

1. Mix PDMS elastomer and hardener at 10:1, defoam in a vacuum chamber, and then pour into the mold (Fig. 8A; **Parts #16**, **#17**, or **#18**).

**Fig. 8:**
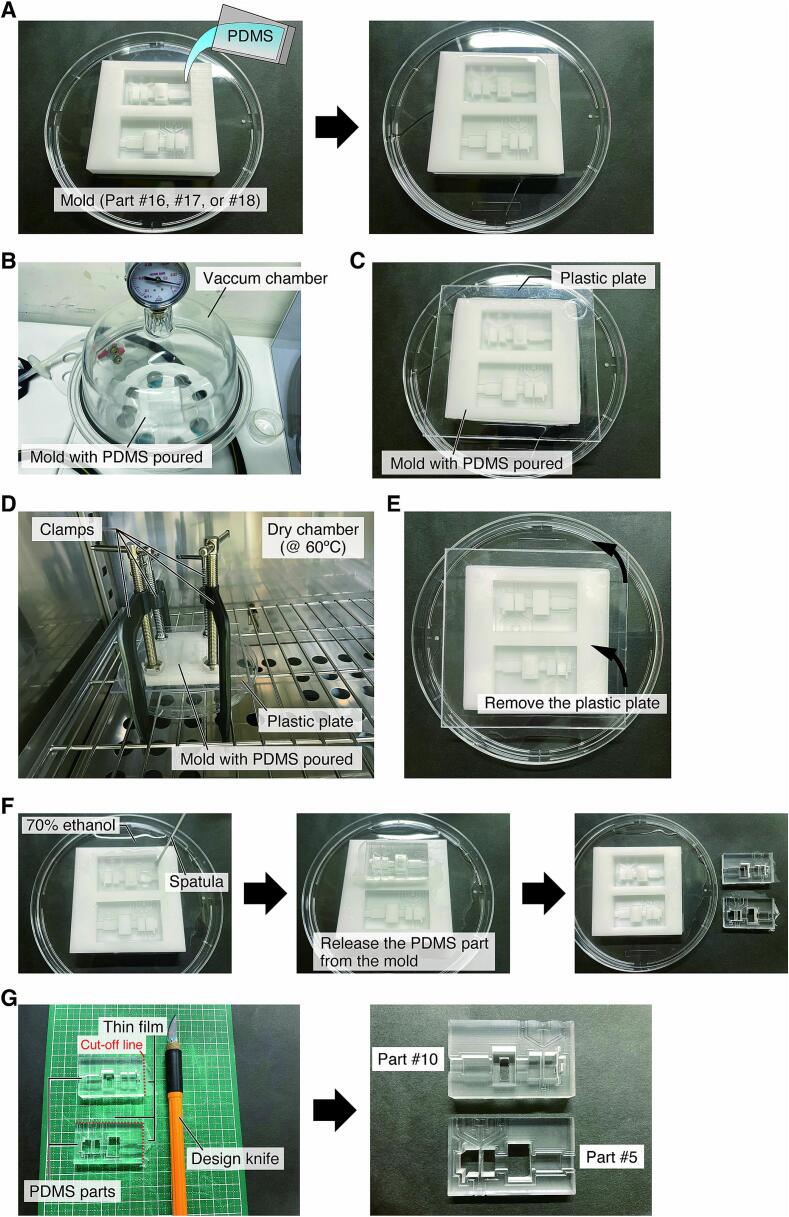
Procedures to fabricate the PDMS parts with soft lithography. A) Pouring a mixture of PDMS elastomer and hardener into the molds. B) Defoaming. C) Shaping the surface of the PDMS poured into the mold by covering it with a plastic plate. D) Curing. E) Removing the plastic plates. F) Removal of PDMS parts from the mold. G) Adjustment of PDMS parts.
2. Perform additional defoaming by placing the mold with the poured PDMS in the vacuum chamber (Fig. 8B).
3. Cover the mold with a plastic plate of polycarbonate or other transparent resin to prevent air bubbles from entering the PDMS (Fig. 8C).
4. Attach the plate to the mold by pressing down evenly with clamps.
5. Place the mold at 60 °C for 12 h to cure the PDMS (Fig. 8D).
6. Remove the plastic plate after confirming that the PDMS has been cured (Fig. 8E).
7. Release the PDMS part from the mold using a spatula while injecting the ethanol into the gap between the part and the mold (Fig. 8F).
8. Cut away the thin film of PDMS that has formed around the part with a knife (Fig. 8G).

#### 5.1.3 Assembly

1. Put a thin layer of PDMS on each bonding surface of the two base bodies (**Parts #5** and **#10**) and glue them together (Fig. 9A). At this point, place the laser holder (**Part #8**) in the designated position between the base bodies in advance, as it cannot be installed later.

**Fig. 9:**
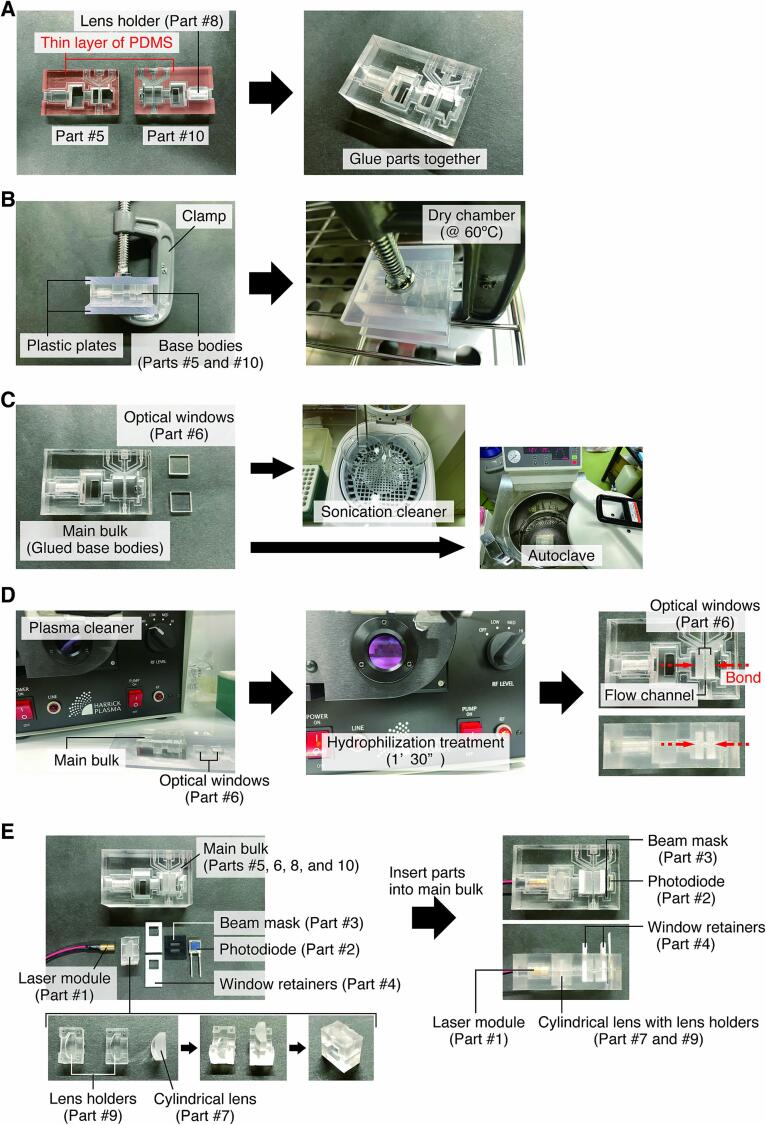
Procedures for assembly of the cell counting module. A) PDMS base body-to-base body gluing. B) Integration of the base bodies (curing). C) Cleaning of main bulk and optical windows. D) Bonding of optical windows to the main bulk by plasma hydrophilization. E) Insertion of optics, electronics, and 3D-printed/CNC-milled parts into the main bulk.
2. Cure the glued surface for 6 h under 60 °C while applying force evenly between the base bodies (main bulk) with plates and a cramp (Fig. 9B).
3. Autoclave the main bulk and sonicate the optical windows (**Part #6**) (Fig. 9C).
4. Dry each part under 60 °C for 12 h.
5. After each part (main bulk and **Part #6**) has been completely dried, apply a temporary hydrophilization treatment for 1 min and 30 s using a plasma cleaner (PDC-32G, Harrick Plasma). Immediately thereafter, attach the plasma-treated surface of the optical windows (**Part #6**) to the flow channel section of the main bulk (Fig. 9D).
6. Insert the laser module, cylindrical lens with holders, photodiode, beam mask, and window retainers into their positions (Fig. 9E).

### 5.2 Preparation of electronic components

A TIA circuit must convert the tiny current output from the photodiode into a voltage that the Arduino can read. The TIA is effective for applications that require sequential reading of weak signals, such as in this case.

**Fig. 10:**
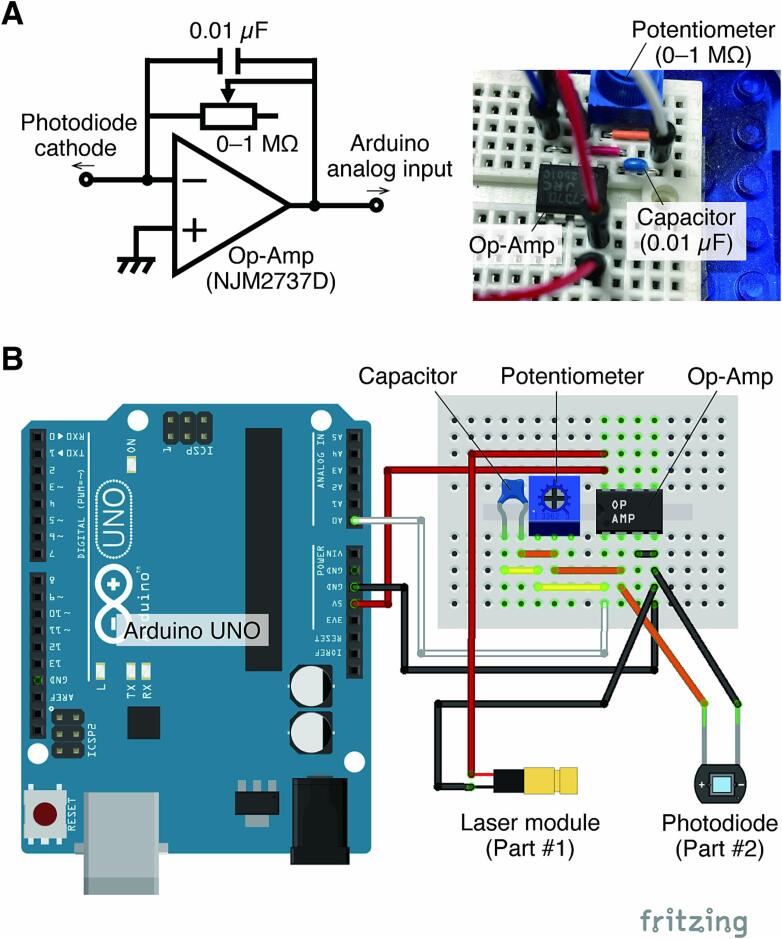
Transimpedance amplifier (TIA) circuit. A) Schematic of the TIA circuit and photograph of the circuit built on a breadboard. B) Configuration to connect the TIA circuit to the Arduino, the laser module, and the photodiode. This configuration diagram was created using Fritzing [19].
Connect the input side of the TIA to the laser module and the output side to the Arduino using jumper wires (Fig. 10B).

Plug and place an op-amp, potentiometer, and capacitor on a breadboard (Fig. 10A). The capacitor is connected in parallel with the feedback resistor (*i.e.*, potentiometer) to filter out high-frequency noise.

### 5.3 Programming of electronic platform

#### 5.3.1 Programming for data storing

An Arduino UNO is used for A/D conversion of the signal voltage, and then the converted digital values are sent to the PC via serial communication. LabVIEW software is used on the PC to receive and store the data. VISA is used as the interface between Arduino and LabVIEW. To acquire data at constant intervals using the Arduino, we need to use its interrupt processing. In this system, a library “TimerOne.h” is installed to acquire the data with a sampling frequency of 4 kHz. The acquired data is stored in a circular buffer, and the accumulated data is sent using a sequential loop function.

1. Extract “Data_Acquisition_Arduino_code.zip”
2. Download and install Arduino IDE.
3. In Arduino IDE, navigate to Sketch > Include Library > Manage Librares.
4. Install the library: “TimerOne”
5. Open “Data_Acquisition_Arduino_code.ino” in Arduino IDE.
6. Connect Arduino Uno to the computer using a cable.
7. In Arduino IDE, navigate to Tools > Board: Select “Arduino Uno” (or respective Arduino platform)
8. In Arduino IDE, navigate to Tools > Port: Select your port
9. Click “Verify”, then click “Upload” and wait for the “upload completed” message.

#### 5.3.2 Programming of signal peak detection and cell counting system

**Fig. 11:**
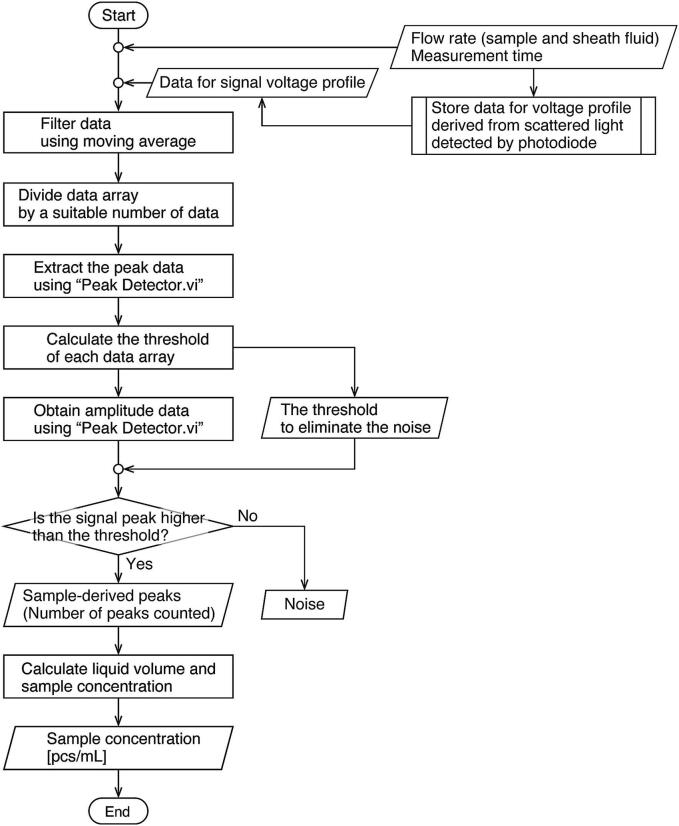
Flowchart of the algorithm for signal peak detection and calculation of sample concentration created using LabVIEW.

The data saved by the program for data storing is read and then filtered by the moving average. Peaks are detected, and their amplitudes are obtained. The information on the amplitudes is displayed in a histogram to visualize the difference between noise and cell-derived values and eliminate noise selectively (Fig. 11). The LabVIEW built-in function “Peak Detector.vi” is used to detect cell-derived peaks. This function applies the least squares method to fit a quadratic function to a given set of data, checks for peaks in the interval based on the obtained function, and, if a peak is present, checks that it exceeds the threshold. This function performs this calculation while shifting data one by one for the data array and outputs information (position, amplitude, etc.) about the peaks in the whole sequence. The appropriate threshold setting is necessary to obtain the amplitude of the peaks accurately. In this program, the threshold is calculated by dividing the data array by a suitable number of data, removing the peaks from the data array for each group, and then averaging the data, considering that the baseline voltage may vary due to disturbances.

## 6 Operation instructions

### 6.1 Operation of cell counting system

1. Connect the Arduino UNO, to which the cell counting module is wired via TIA, to the PC with a USB cable and start up the system (Fig. 12A).

**Fig. 12:**
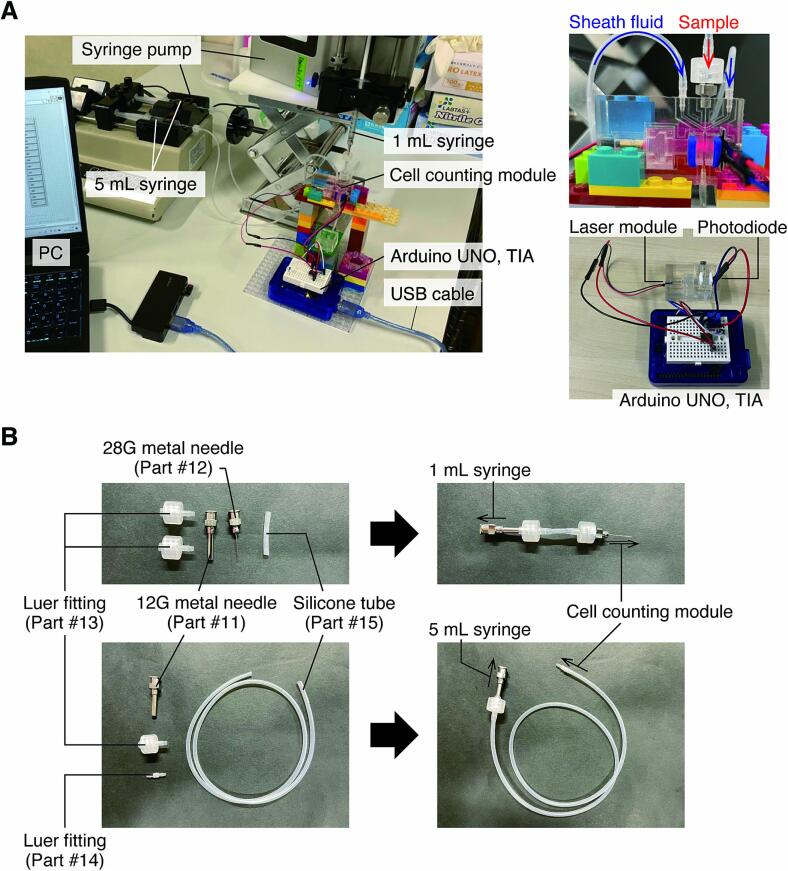
Overall configuration of cell counting system. A) Photographs of the cell counting system in operation. B) Details of how to connect silicone tubes, metal needles, and lure fittings.
2. Prepare the cell suspension to be measured.
3. Fill a 1 mL syringe with 100 µL of cell suspension and two 5 mL syringes with 5 mL of sheath fluid.
4. Set the syringes to be filled with a sample and sheath fluid into the pumping device. Use of a syringe pump is recommended (*e.g.*, Legato 110 and KDS270, KD Scientific).
5. Connect each syringe to the module using a silicone tube (**Part #15**; Fig. 12B).
6. Set the flow rate of each syringe pump appropriately (*e.g.*, sample: 1 µL/min, sheath fluid: 30 µL/min x 2) and inject the sample and sheath fluid into the flow channel of the cell counting module.
7. Save the signal output from the photodiode with the program for data storing.
8. Calculate the sample concentration in the program for signal detection and cell counting system after the data has been saved. At this time, noise is appropriately eliminated from the histogram of peak amplitudes.
9. When operating the system consecutively, include a wash cycle with enough blank solution (*e.g.*, ultrapure water or PBS) between measurements of different samples to avoid cross-contamination.

### 6.2 Maintenance of cell counting module

The cell counting module can be operated repeatedly by performing the appropriate maintenance as described below after each use.

1. Carefully rinse the silicone tubes (luer fittings), the needles, and the flow channel of the cell counting module with ultrapure water. It is recommended to use a 5 mL syringe at this point to flush the ultrapure water into them.
2. Wipe off any dust on the outside of the module with 70 % ethanol.
3. Dry the entire kit, including the cell counting module, until the next operation.
4. The red laser module (**Part #1**) has a limited life of several hundred hours. If the module is worn out, replace it with a new one.
5. Other electronic and optical components must also be replaced if they malfunction or break due to repeated use. They can be replaced in the same way as described in “**5.1. Hardware preparation**”.

## 7 Validation and characterization

To characterize the performance of the cell counting module, we performed two types of validation: one was a comparison with a commercially available hemocytometer (*i.e.*, counting accuracy), and the other was sorting objects of different diameters with the fabricated module (*i.e.*, counting resolution).

### 7.1 Validation of cell counting accuracy

**Fig. 13:**
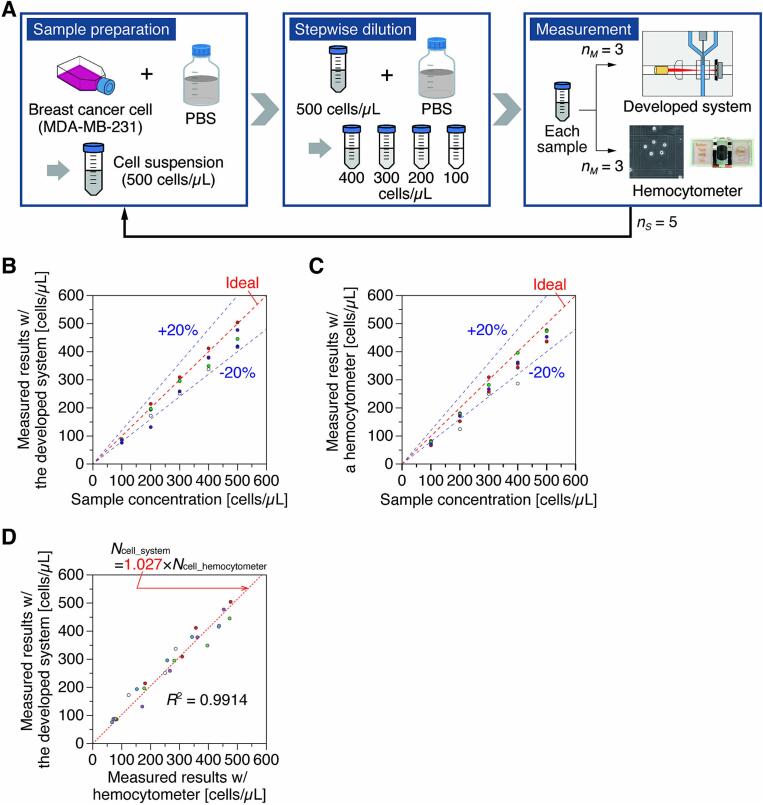
Validation of counting accuracy. A) Procedures for cell sample preparation and measurement. Measured results with B) the developed cell counting system or C) a conventional hemocytometer. D) Comparison of results measured with each device.

Cell suspensions with multiple concentrations were prepared, and each was measured using the developed cell counting module and a commercial hemocytometer (Bürker-Türk type, 03–303-1, Erma). Human breast adenocarcinoma cell line (MDA-MB-231; AKR-201, Cell Biolabs) was cultured in culture medium (Dulbecco’s modified eagle medium (DMEM); 31600–034, Gibco, Thermo Fisher Scientific) to 90 % confluence, detached using 0.25 % trypsin-EDTA (25200–072, Gibco), collected with culture medium, centrifuged (450 *g*, 5 min), and the supernatant was removed. After removing the supernatant, the cell concentration was adjusted using phosphate-buffered saline (PBS; 05913, Nissui Pharmaceutical), and five different concentrations of cell suspension were prepared by stepwise dilution (Fig. 13A). The cell suspension concentrations used for validation were 100, 200, 300, 400, and 500 cells/µL. The flow rates of the sample (cell suspension) and the sheath solution were set to 1 µL/min and 30 µL/min, respectively. For both instruments, measurements were performed three times with the same suspension, and the average value was used as the measured concentration. The experiments were performed five times to verify the measurement performance of the developed module.

The cell counting results using the prepared cell suspensions at five different concentrations showed that the module (Fig. 12B) and the hemocytometer (Fig. 13C) had slightly lower values than the ideal concentration values. Still, both instruments could measure within approximately 20 % of the ideal concentration value. Comparing the measurement results of the two instruments, we can see that the slope of their regression lines is 1.027 and the correlation coefficient is *R*^2^ = 0.9914 (Fig. 13D). Thus, we can conclude that the measurement results of the two instruments show almost equal values, which confirms that the accuracy of the developed module is equivalent to that of commercial hemocytometer. The reason why the measured values for both instruments were lower than ideal is due to human error (loss or dilution blurring) during sample adjustment. This can happen even with high-end instruments such as flow cytometers. On the other hand, the module can be used for routine cell counting without significant problems.

### 7.2 Validation of cell counting resolution

**Fig. 14:**
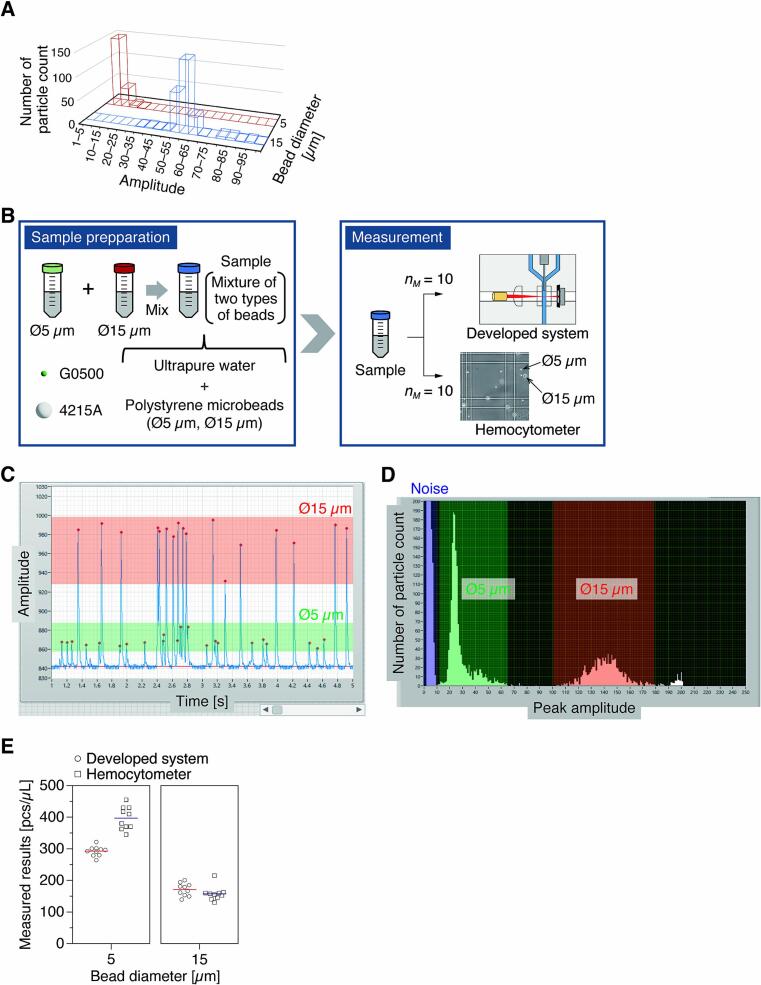
Validation of counting resolution. A) Differences in the peak amplitude values derived from scattered light intensity depending on the bead diameter. B) Procedures for cell sample preparation and measurement. Measured results with C) Waveform data of the scattered light intensity derived from the beads with two different diameters. D) Histogram created based on the total peak amplitude values. E) Results of simultaneous measurement for the beads with two different diameters using the developed system.

A critical issue for application to cell sorting in the future is the ability to isolate cells of different sizes and the presence of dead cells in a sample. We then examined whether it is possible to sort out peaks derived from target cells in a sample by the intensity of forward scattered light (*i.e.*, peak amplitude). A suspension containing microparticles with two distinctly different diameters was prepared, and the concentration of each particle was measured. We used green aqueous fluorescent particles (G0500, Thermo Fisher Scientific; Note: Use of fluorescent labels is not mandatory) with a diameter of 5 µm and polystyrene monosized particles (4215A, Thermo Fisher Scientific) with a diameter of 15 µm. We first adjusted each particle suspension individually to obtain the scattered light intensity, determined the reference values of their peak amplitudes (Fig. 14A), and then measured them in the mixed sample. A mixed sample was prepared by setting and adjusting the concentration of the two types of particles to a moderate concentration in the range of 100–500 particles/µL. The flow rates of the sample and the sheath fluid were set to 1 µL/min and 30 µL/min, respectively. We evaluated the resolution of the developed module by measuring the concentrations ten times with both instruments (Fig. 14B).

The waveform data of the scattered light intensity in the measurement showed that differences in the diameter of each particle could be detected from the peak amplitude values (Fig. 14C). We could also confirm that the histogram created based on the total peak amplitude values showed a distribution with two bumps (Fig. 14D). Hence, we determined the concentration of each particle by setting a threshold value at the boundary of these bumps (Fig. 14E). A similar trend was observed for the module and the hemocytometer, suggesting that it is possible to detect particles of different diameters using the module. However, the module showed a tendency for the concentration of particles with a diameter of 5 μm to be lower. This is because the peak voltage values obtained from 5 μm diameter particles are small in amplitude and difficult to sort from noise. Therefore, an appropriate threshold setting that allows noise elimination plays an important role in cell sorting in the module.

### CRediT authorship contribution statement

**Takanobu Takenouchi:** Writing – review & editing, Writing – original draft, Visualization, Validation, Methodology, Investigation, Conceptualization. **Yuta Iijima:** Writing – review & editing, Validation, Methodology, Investigation. **Kazuyo Ito:** Writing – review & editing, Validation, Methodology. **Daisuke Yoshino:** Writing – review & editing, Writing – original draft, Visualization, Validation, Supervision, Resources, Methodology, Funding acquisition, Conceptualization.

## Declaration of competing interest

The authors declare that they have no known competing financial interests or personal relationships that could have appeared to influence the work reported in this paper.
